# Specificity of two monoclonal antibodies against a synthetic glycopeptide, an analogue to the hypo-galactosylated IgA1 hinge region

**DOI:** 10.1007/s40620-014-0118-4

**Published:** 2014-07-19

**Authors:** Yoshiyuki Hiki, Hideo Hori, Kouichiro Yamamoto, Yoshihiro Yamamoto, Yukio Yuzawa, Nobuya Kitaguchi, Kazuo Takahashi

**Affiliations:** 1 Department of Clinical Engineering, School of Health Sciences, Fujita Health University, 1-98 Dengakugakubo, Kutsukake-cho, Toyoake, Aichi 470-1192 Japan; 2 Department of Nephrology, School of Medicine, Fujita Health University, 1-98 Dengakugakubo, Kutsukake-cho, Toyoake, Aichi 470-1192 Japan

**Keywords:** IgA nephropathy, IgA1 hinge region, *N*-acetylgalactosamine, Galactose, Monoclonal antibody

## Abstract

Increased levels of hypo-galactosylated immunoglobulin (Ig)A1 (HG-IgA1) in IgA nephropathy (IgAN) have been detected using a *Helix aspersa* agglutinin lectin enzyme-linked immunosorbent assay (ELISA). In this study, we developed monoclonal antibodies to evaluate the HG-IgA1 in IgA nephropathy, aiming to gain a more consistent and reproducible assay. As an analogue to the HG-IgA1 hinge region, a 19 mer synthetic peptide with five GalNAc (sHGP) residues at positions 4, 7, 9, 11 and 15 [VPST(GalNAc)PPT(GalNAc)PS(GalNAc)PS(GalNAc)TPPT (GalNAc)PSPS-NH2] was synthesized. Two monoclonal antibodies against sHGP (35A12 and 44H8) that reacted with human IgA were developed. Also, their reactivities to serum IgA from IgAN patients (*n* = 49), patients with other forms of kidney diseases (OKD, *n* = 48), and healthy controls (HC, *n* = 41) were evaluated using ELISA assays. The binding levels of the two monoclonal antibodies against serum IgA were significantly higher (all comparisons, *p* < 0.0001, Steel–Dwass non-parametric test) in IgAN patients compared to HC and OKD patients. In each individual, there was a close correlation of IgA binding levels between 35A12 and 44H8 (*R* ^2^ = 0.737). These results indicate that the monoclonal antibodies recognize similar epitopes in HG IgA1, which is found predominantly in IgAN patients. The developed antibodies are proposed as a clinically useful tool for IgAN screening.

## Introduction

Immunoglobulin (Ig)A nephropathy (IgAN) diagnosis is currently confirmed using an invasive method of renal biopsy, as the presence of predominant mesangial IgA deposits is the gold standard for diagnosis. Several candidates of serum and urinary markers for identifying IgA nephropathy have been proposed, such as anti-IgA antibody [1, 2] and anti-IgA1 hinge peptide antibody [3, 4]. However, they exhibit insufficient specificity for clinical application to identify IgAN.

There is increasing evidence supporting the involvement of aberrantly glycosylated IgA1 in the pathogenesis of IgAN [5–12]. It has been suggested that the presence of truncated *O*-glycans with an exposed GalNAc residue is more common on the IgA1 of IgAN patients. Previously, *O*-glycans in the hinge region of IgA1 were evaluated with an enzyme-linked immunosorbent assay (ELISA) using the *Helix aspersa* agglutinin (HAA) lectin, which specifically binds to GalNAc residues [13, 14]. The results indicated that there is increased binding of HAA to serum IgA1 from IgAN patients. In a previous study, we produced a rabbit polyclonal antibody against a synthetic IgA1 hinge peptide containing five GalNAc residues as an analogue of galactose-deficient IgA1 hinge region. The polyclonal antibody was capable of binding to serum IgA1 but poorly identified IgAN [15]. In this study, therefore, we attempted to develop monoclonal antibodies that specifically recognize hypo-galactosylated IgA1, which is found predominantly in IgAN patients.

## Materials and methods

### Patients and test sera

The study was approved by the ethical committee (No.14-053) of Fujita Health University in accordance with the Helsinki Declaration. Forty-nine patients with biopsy-proven IgAN were examined. An additional 48 patients with other kidney diseases (OKD) and 41 healthy subjects (HC) were enrolled in the study as disease and healthy controls, respectively. The number of OKD patients and their disease details are reported in Table 1. The HC subjects were selected from healthy individuals who were gender- and age-matched to the IgAN patients. All sera samples were stored at −80 °C until use.

**Table 1:** Itemization of patients with other kidney diseases (OKD)

Disease	Number of patients
Lupus nephritis	22
Anti-neutrophil cytoplasmic autoantibody associated nephritis	11
Membranous nephropathy	4
Minimal change nephrotic syndrome	2
Focal segmental glomerulosclerosis	3
Nephrosclerosis	2
Amyloidosis	2
Cholesterol crystal embolism	1
Diabetic nephropathy	1
Total	48

### Antigen

A 19 mer synthetic peptide with five GalNAc (sHGP) residues at positions 4, 7, 9, 11 and 15 [VPST(GalNAc)PPT(GalNAc)PS(GalNAc)PS(GalNAc)TPPT(GalNAc)PSPS-NH2] was purchased from the Peptide Institute, Inc. (Osaka, Japan). The GalNAc glycosylation sites were determined according to Mattu et al. [16], who found that GalNAc residues are frequently located at these sites in the human IgA1 hinge region. The purity and molecular weight were confirmed by high performance liquid chromatography (HPLC) and matrix-assisted laser desorption ionization time of flight mass spectrometry (MALDI-TOF-MS). For immunization, the sHGP was conjugated with keyhole limpet hemocyanin (KLH; Sigma Chemical Company, St. Louis, MO, USA).

### Monoclonal antibodies

Monoclonal antibodies against sHGP-KLH were produced by Tomiyama Laboratory Co. Ltd. (Tokyo, Japan) using conventional procedures. Briefly, 100 μg of sHGP-KLH was subcutaneously injected into a BALB/cA mouse (6-week-old female, CLEA Japan, Inc. Shizuoka, Japan) three times, with 2 weeks between injections. Three days after the final immunization, spleen and lymph nodes were collected from the mouse under general anesthesia. The lymphocytes isolated from the immunized mouse were mixed with myeloma cells at a ratio of 5:1. Initially, the mixed cells were gently suspended in 1 ml of 50 % polyethylene glycol (PEG) solution (PEG 1500, Roche Diagnostic Corp., Indianapolis, USA) for cell fusion, and subsequently resuspended in RPMI-1640 (Gibco Life Technologies, Grand Island, NY, USA) containing 15 % FBS (Biowest, Nuaille, France) and hypoxanthine aminopterin thymidine (HAT) supplement (Gibco Life Technologies). The cells were incubated in a 96-well culture plate for 1 week in a CO_2_ incubator for cell proliferation. Antibody screening was performed using ELISA to select culture supernatants, producing antibodies that reacted with sHGP and serum IgA, but not with synthetic hinge peptide without GalNAc residues (sHP). After cloning of the hybridoma cells, hybridoma implantation was performed and ascites were collected. As a result, six monoclonal antibodies [35A12 (IgG subclass: IgG3κ), 35H10 (IgG1κ), 38A7 (IgG1κ), 43C5 (IgG2bκ), 44H8 (IgG1κ), and 54B9 (IgG2bκ)] were raised as candidates to identify serum hypogalactosylated IgA1. The IgG fractions were isolated from ascites using conventional ion-exchange chromatography. From the pilot study using a small number of IgAN and HC sera, we ultimately selected two monoclonal antibodies, 35A12 and 44H8, as convincing markers of IgAN.

**Fig. 1:**
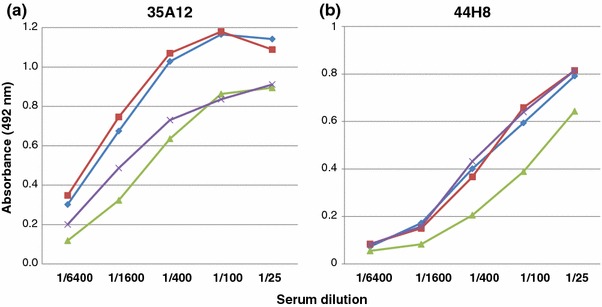
Dose response curves of 35A12 (**a**) and 44H8 (**b**) monoclonal antibodies in four serum samples. Neuraminidase-treated human serum samples (three IgAN and one HC) were serially diluted from 1/25 to 1/6,400

We constructed dose–response curves for IgA binding to the monoclonal antibodies, 35A12 and 44H8, using serially diluted (1/25–1/6,400) neuraminidase-treated human serum samples (Fig. 1a, b).

### ELISA assay

Serum IgA binding levels to each monoclonal antibody were compared among the IgAN, OKD and HC groups using the following ELISA assay. A 50 µl aliquot of 0.05 M bicarbonate buffer (pH 9.6) containing 10 μg/ml of monoclonal antibody was added to the wells of a 96-well polystyrene microtiter plate (Corning Inc., Corning, NY, USA) and incubated overnight at 4 °C. The plates were washed with 0.01 M phosphate buffered saline (PBS)—0.05 % Tween-20 (pH 7.4, PBST) three times at each step. Coated plates were blocked with 1 % bovine serum albumin (BSA; Sigma Chemical Company) in PBS for 1 h at room temperature (RT). The coated plates were stored at 4 °C.

Removal of terminal neuraminic acid from the IgA1 glycans, leaving terminal GalNAc residues, was accomplished by incubating serum samples with five volumes of 0.01 M acetate buffer (pH 5) containing 1.5 mU/ml neuraminidase (from *Vibrio cholerae*, Roche Diagnostic Corp., Indianapolis, IN, USA) for 4 h at 37 °C in micro-tubes (1.5 ml; Crystalgen Inc., Commack, NY, USA). The neuraminidase-treated samples were optimally diluted with PBST containing 1 % bovine serum albumin (BSA), and a 50 µl/well aliquot was added to the coated wells and incubated overnight at 4 °C. After three washings with 250 µl of PBST, a 100 μl aliquot of horseradish peroxidase (HRP)-labeled goat anti-human IgA (1:5,000 dilution, IgG Fab fraction; MBL Co., LTD, Nagoya, Japan) was added to each well. Following a further 2 h incubation at 25 °C, the wells were washed 5 times with 250 µl of PBST, and the signal was detected with 100 µl of *O*-phenylenediamine (0.5 mg/ml Wako Pure Chemical Industries, LTD., Osaka, Japan)—H_2_O_2_ (0.1 %) in 0.1 M citrate phosphate buffer. The colour reaction was stopped with 100 µl of 1 M sulfuric acid, and absorbance at 492 nm was measured.

### Data analyses and statistics

To correct for variance between experimental conditions, results were expressed as standard deviation units (SDU), which were calculated as follows: SDU of each sample = (sample absorbance − mean absorbance of healthy controls)/standard deviation of healthy controls, where the mean and SD values of HC calculated in each experiment were used.

The statistics software package JMP10 (SAS Institute Inc., Cary, NC, USA) was used to analyze the data. The Kruskal–Wallis non-parametric test was used to identify significant differences in binding levels of the two monoclonal antibodies among IgAN, OKD, and HC groups (both antibodies *p* < 0.0001). The binding levels were then compared between one group and another in the three groups using the Steel–Dwass non-parametric test. Differences were regarded as statistically significant at a *p* value <0.05.

## Results

### Comparisons of antibody reactivities among IgAN, OKD and HC groups

**Fig. 2:**
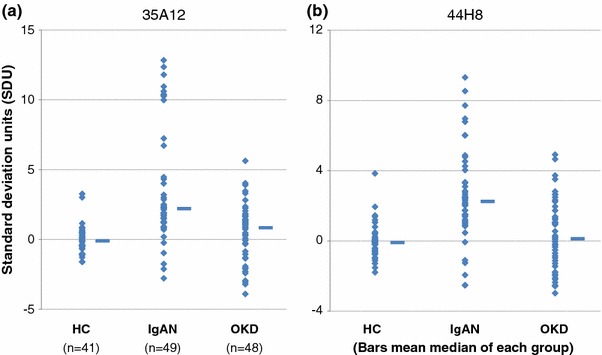
Comparisons of IgA levels bound to two monoclonal antibodies in the HC, IgAN and OKD groups (**a** 35A12, **b** 44H8). **a** The IgA binding level of 35A12 monoclonal antibody in the IgAN group was significantly increased compared to the OKD and HC groups (both *p* < 0.0001). There was no significant difference between HC and OKD (*p* = 0.113). **b** The IgA binding level of 44H8 antibody in the IgAN groups was significantly increased compared to the HC and OKD groups (both *p* < 0.0001). There was no significant difference between HC and OKD (*p* = 0.831)

The IgA binding levels of the two monoclonal antibodies in the IgAN group were similarly increased compared to levels in the OKD and HC groups. With the 35A12 monoclonal antibody, significant differences were observed between IgAN vs. HC, and IgAN vs. OKD (both *p* < 0.0001, Steel–Dwass non-parametric test, Fig. 2a); however, no difference was found between HC vs. OKD (*p* = 0.113). With the 44H8 antibody, a significant increase was observed in the IgAN group compared to the HC and OKD groups (both *p* < 0.0001, Fig. 2b). There was no significant difference between HC and OKD (*p* = 0.831).

### Correlation between B35A12 and 44H8 antibodies

**Fig. 3:**
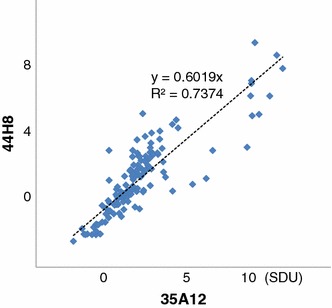
Correlation between 35A12 and 44H8 IgA binding levels. There was a strong correlation between the results obtained for the 35A12 and 44H8 antibodies (*R* ^2^ = 0.737), suggesting that the two monoclonal antibodies recognize a similar epitope of sHGP, and that the results of the IgA binding levels are reproducible

The IgA binding levels of 35A12 and 44H8 antibodies were strongly correlated in individual samples (*R* ^2^ = 0.737) (Fig. 3).

### Receiver operating characteristic (ROC) curves

**Fig. 4:**
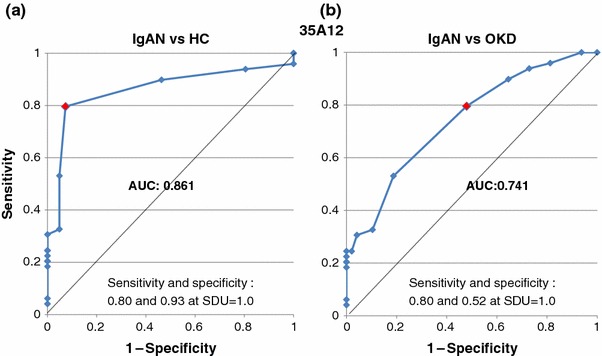
Receiver operating characteristic (ROC) curves of the 35A12 antibody between the IgAN and HC groups, and the IgAN and OKD groups. The AUC values of IgAN patients for 35A12 against HC (**a**) and OKD (**b**) were 0.861 and 0.741, respectively

**Fig. 5:**
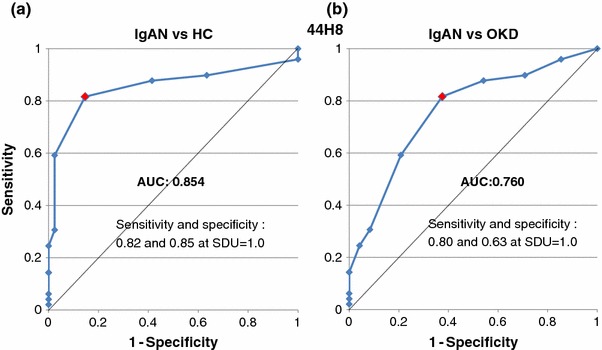
Receiver operating characteristic (ROC) curves of the 44H8 antibody between the IgAN and HC groups, and the IgAN and OKD groups. The AUC values of IgAN patients for 44H8 against HC (**a**) and OKD (**b**) were 0.854 and 0.760, respectively

The areas under the curve (AUC) calculated from the ROC curves for the IgAN group are summarized in Table 2. The AUC values of IgAN patients for 35A12 against HC (Fig. 4a) and OKD (Fig. 4b) were 0.861 and 0.741, respectively. The AUC values for 44H8 were 0.854 (Fig. 5a) and 0.760 (Fig. 5b) for HC and OKD, respectively.

**Table 2:** Summary of AUC of ROC curves for each monoclonal antibody

Monoclonal antibody	vs. Healthy controls	vs. Other kidney diseases
35A12	0.861	0.741
44H8	0.854	0.760

## Discussion

Human IgA1 has a unique hinge structure due to the presence of mucin-like *O*-linked oligosaccharides [16]. The core peptide consists of a proline-, serine-, and threonine-rich amino acid sequence in which the serine and threonine residues are *O*-glycosylated with structures consisting of neuraminic acid, galactose and *N*-acetylgalactosamine, with microheterogeneity observed. In the past two decades, it has been demonstrated that certain abnormalities exist in the *O*-glycan structures of the IgA1 hinge region in IgAN. Although these abnormalities have been suggested to be due to hypo-glycosylation (especially hypo-galactosylation), the precise structure of these glycans is not yet clear. The HAA-assay was capable of demonstrating a significant increase in exposed GalNAc residues in IgAN samples. In other words, IgAN patients had fewer Gal residues in the IgA1 hinge region. However, the relatively weak binding affinity of lectins to the corresponding carbohydrates made the assay unstable and insufficiently reproducible.

Previously, we developed a polyclonal antibody using a synthetic hinge glycopeptide [15]. The polyclonal antibody bound more strongly to asialo/agalacto-IgA1 than untreated and asialo-IgA1, suggesting that the antibody recognized hinge peptides containing GalNAc residues. Further, the binding levels of the polyclonal antibody were closely related to those observed using the HAA ELISA in the same patient sera (*R* ^2^ = 0.5964), with IgAN binding significantly increased compared to the OKD and HC groups. However, the polyclonal antibody was far inferior to the monoclonal antibodies introduced in this study.

In this study, we developed two monoclonal antibodies against a synthetic hinge glycopeptide, which is regarded as a model of hypo-galactosylated IgA1 hinge protein. The increased antigenicity against the antibody in IgAN suggested that IgAN patients have hypo-glycosylated IgA1 with exposed GalNAc residues on the hinge core peptide.

It is unlikely that the two monoclonal antibodies are currently clinically applicable as substitutes for renal biopsy. Both monoclonal antibodies exhibited insufficient specificity for differentiating IgAN from other kidney diseases. However, as the sensitivities were acceptable for clinical applications, it may be possible to utilize them as non-invasive tools for IgAN screening. It is expected that the development of a highly specific antibody could replace the invasive renal biopsy method for diagnosing IgAN.

## Abbreviations

IgA: Immunoglobulin A
IgAN: IgA nephropathy
GalNAc: *N*-acetylgalactosamine
sHGP: Synthetic hinge glycopeptide
HC: Healthy controls
OKD: Other forms of kidney diseases
HAA: *Helix aspersa* agglutinin
HPLC: High performance liquid chromatography
MALDI-TOF–MS: Matrix-assisted laser desorption ionization time of flight mass spectrometry
KLH: Keyhole limpet hemocyanin
AUC: Area under the curve
ROC: Receiver operating characteristic
